# Editorial

**Published:** 1849-12

**Authors:** 


### THE MEDICAL EXAMINER.

PHILADELPHIA, DECEMBER, 1849.

### TO OUR READERS.

With the present number is completed the 12th volume of the Medical Examiner. A glance at its past history, exhibits it, at this time, in a more prosperous condition, and with a more extended circulation than at any previous period since its commencement. The promises of continued and increased collaboration, together with the commendations of, perhaps, too partial friends, warrant the hope of still better things, and justify the belief that future endeavors to supply to its patrons food "good and convenient for them" will not be unsuccessful.

To the numerous friends who have so kindly aided the Editors, and to whose exertions the success of the Examiner is mainly owing, their warmest thanks are offered. The large amount of original matter that has been presented to its readers during the past year, affords ample evidence of the steadiness and reality of that support.

With the present number also ceases the official connection of Dr. Tucker, recently appointed Professor of Practice in Hampden Sidney College. The remaining Editor, on whom now devolves the sole responsibility of the Journal, cannot permit the present occasion to pass without bearing witness to the assiduity and zeal with which the labors of his colleague were ever performed, and to the pleasure that was derived from daily and intimate association with him. While he regrets his own loss, he congratulates his Southern friends on their gain.

The course of the Examiner will continue to be, as heretofore, *independent*, unbiassed either by local influence or sectional prejudices,—its great aim being, not to subserve any selfish ends, but to aid to the utmost of its ability, in building up and maintaining *American Medical Literature*.

### OBITUARY.

Dr. George Massenburg, one of the resident physicians at the *Philadelphia Hospital*, Blockley, died at that institution, Nov. 13th, of typhus fever. Dr. Massenburg was a native of Hampton, Va., and a gentleman of fine talents, superior attainments, and most exemplary character.

His death is a source of deep regret to all the officers of the institution with which he was connected, as well as to a large circle of friends by whom he was greatly beloved.

PHILADELPHIA HOSPITAL, BLOCKLEY.

Dr. N. D. Benedict has resigned his position as chief resident physician to the Philadelphia Hospital, Blockley, and accepted that of Superintendent of the N. Y. State Lunatic Asylum, at Utica, made vacant by the death of Dr. Brigham. Dr. Benedict's long devotion to the treatment of the Insane, eminently qualifies him to fulfil successfully the duties of the responsible office to which he has been appointed. Dr. W. S. Haines succeeds him ; a good selection.

ST. JOSEPH'S HOSPITAL, PHILADELPHIA.

Dr. J. H. B. McClellan has been appointed one of the surgeons of this Institution. The Hospital Staff now consists of Drs. W. E. Horner, H. H. Smith, and McClellan, Surgeons, and Drs. S. Jackson, A. Stillé, and W. H. Keating, Physicians.

CRYPTOGAMOUS ORIGIN OF CHOLERA.

The following are the conclusions arrived at by a committee of the London College of Physicians, appointed to examine this subject:

The "Cholera fungi" do not exist in the waters of a large number of the districts in which cholera prevails.

The "Cholera fungi" cannot, by the most careful examinations, be detected in the air of many rooms inhabited by cholera patients.

"Cholera fungi" are constantly to be found in the stools passed by patients laboring under other diseases than cholera.

"Cholera fungi" are occasionally to be found in healthy stools.

"The bodies which have been called 'different forms of the development of the cholera fungus,' are in quality the most dissimilar in their origin and chemical constitution."

MEDICAL DEPARTMENT, U. S. ARMY.

At the recent meeting of the Board of Examiners, held in this city, the following gentlemen were approved, and assigned rank as Assistant Surgeons, in the order of their names. Isaac L. Adkins, *Delaware;* Robert O. Abbott, *Pennsylvania;* Thomas M. Getty, *Virginia;* David L. Magruder, *Virginia;* Wm. J. H. White, *District of Columbia;* Rodney Glisau, *Maryland;* Elisha P. Langworthy, New York.

*Assistant Surgeon,* James Simons, was found qualified for promotion by the same board.

